# GRANDPA: GeneRAtive network sampling using degree and property augmentation applied to the analysis of partially confidential healthcare networks

**DOI:** 10.1007/s41109-023-00548-5

**Published:** 2023-05-11

**Authors:** Carly A. Bobak, Yifan Zhao, Joshua J. Levy, A. James O’Malley

**Affiliations:** 1 Department of Biomedical Data Science, Dartmouth College, Hanover, NH USA; 2 The Dartmouth Institute for Health Policy and Clinical Practice, Dartmouth College, Hanover, NH USA; 3 Research Computing, Dartmouth College, Hanover, NH USA; 4 Department of Pathology and Laboratory Medicine, Dartmouth College, Hanover, NH USA; 5 Department of Dermatology, Dartmouth College, Hanover, NH USA; 6 Department of Epidemiology, Dartmouth College, Hanover, NH USA

**Keywords:** Generative graphs, Simulation, Property graphs, Confidentiality, Healthcare data privacy

## Abstract

Protecting medical privacy can create obstacles in the analysis and distribution of healthcare graphs and statistical inferences accompanying them. We pose a graph simulation model which generates networks using degree and property augmentation and provide a flexible R package that allows users to create graphs that preserve vertex attribute relationships and approximating the retention of topological properties observed in the original graph (e.g., community structure). We illustrate our proposed algorithm using a case study based on Zachary’s karate network and a patient-sharing graph generated from Medicare claims data in 2019. In both cases, we find that community structure is preserved, and normalized root mean square error between cumulative distributions of the degrees across the generated and the original graphs is low (0.0508 and 0.0514 respectively).

## Introduction

Graph theory and network analysis play vital roles in the study of complex relationships relevant to biology, disease, and healthcare delivery. Common structures of these networks include network vertices as genes, proteins, physicians, hospitals, etc. where edges often represent co-occurrence or correlation (Fernández-Peña et al. 2022; Koutrouli et al. 2020; Van Der Wijst et al. 2018; Infante et al. 2020). Such networks frequently contain multiple characteristics on the vertex level (referred to as attributes or properties) which contain information on the individual vertices, whereas characteristics on the edge level contain information between two vertices. For instance, network analysis has been used to study the diffusion of COVID-19 infection through hospital employees (Garzaro et al. 2020), to uncover new findings in the pathogenesis of Tuberculosis (Bobak et al. 2022), and are frequently used to study and propose novel cancer biomarkers (Kosvyra et al. 2021).

Networks for healthcare applications are often constructed using confidential medical data. Social healthcare networks can be constructed from insurance claims, medical records, and electronic health records (Landon et al. 2018; Cusumano-Towner et al. 2013; O’Malley et al. 2020; Escribe et al. 2022). Biological networks have traditionally been constructed by observing relationships between potential biomarkers (genes, proteins, transcription factors, etc.) across an entire cohort, but in the era of personalized medicine, networks constructed from one individual’s personal biological information have been proposed (Koutrouli et al. 2020; Van Der Wijst et al. 2018; Infante et al. 2020). In both social and biological networks, necessary privacy and confidentiality precautions, including storage of network data on secure servers and limitations to thoroughly vetted computational tools of such data, can create obstacles in the analysis and distribution of healthcare networks (Clayton et al. 2019; Sathanur et al. 2017; Bonomi et al. 2020). Thus, there is a need to generate novel networks which maintain the structural properties of healthcare networks without compromising or distributing confidential medical data.

Among the many models and approaches to generate networks that have been developed (Barabási and Albert 1999; Erdos and Rényi 1984; Watts and Strogatz 1998; Hunter et al. 2008; Csardi 2014; Chandrasekhar and Jackson 2018), most emphasize simulating the overall network topology and rarely consider the role of vertex attributes. Exponential random graph models (ERGMs) are among the more flexible options (Hunter et al. 2008), although have been noted to have unstable parameter estimation on large networks and those with dyadic dependent terms (Chandrasekhar and Jackson 2018). In many cases, the information stored in the vertex attributes is directly related to the application of interest—for instance, (1) studies of homophily (i.e., do vertices with similar attributes connect more frequently than expected after conditioning on other network features and properties), (2) heterophily (i.e., do differing vertices connect more frequently than expected), and (3) studies examining network characteristics related to community or clustering, the spread of health technologies, etc. All of these studies would require the preservation of vertex-level information. To address this, Kim and Leskovec (2010) introduced the Multiplicative Attribute Graph (MAG), which assumes that vertex attributes are indicative of latent graph structure, and seeks to generate graphs using such structure. Building on this, Pfeiffer et al. introduced the Attributed Graph Model (ATG) which similarly seeks to generate graphs from attribute structure, but uses an accept-reject sampling procedure to do so Pfeiffer et al. (2014). It has been previously posited that both vertex similarity (homophily) and vertex popularity (degree) should be used to generate networks reflective of those observed in the real world (Papadopoulos et al. 2012). In response, Sathanur et al. introduced the Property Graph Model (PGM) which calculates the joint label assignment probabilities for vertices and joint distribution probabilities over pairs of vertices for the edges. They then ’augment’ their original label categories by partitioning the degree distribution into bins and assigning each vertex a label corresponding to the bin its degree is in. They demonstrate that this algorithm is scalable (linear in the number of edges), preserves attribute relationships, and better represents degree structure (Sathanur et al. 2017).

Community detection in networks has long been established as an important component of network study (Newman 2006; Cherifi et al. 2019; Fortunato and Newman 2022). In real-world networks, nodes naturally organize into clustered communities or modules and these communities are often meaningful units for analysis. For instance, community structure is often considered in biological networks and facilitates the study of how groups of biomarkers work in tandem to control biological processes (Sah et al. 2014; Alcalá-Corona et al. 2016; Langfelder and Horvath 2008; Calderer and Kuijjer 2021). As well, community structure of physicians at hospitals has been associated with patient readmission rates and overall hospitalization costs (Uddin et al. 2015). Hence, graph generating algorithms which preserve community structure alongside vertex attributes are necessary to simulate networks which can be analyzed to study healthcare phenomena. In terms of recovering the community structure in real-world graphs, in prior work community detection algorithm have been found to be useful. For example, Karrer and Newman (2010) showed that stochastic blockmodels incorporating heterogeneity of node degree could generate benchmark graphs with similar degree distribution compared to real graphs. Also, Kirkley and Newman (2022) proposed a method, which outputs a representative set of community partitions and could reveal the multimodal community structure presented in real-world graphs.

In this work, we created an R package to allow for the easy implementation of the PGM generative graphs in the R programming language, favored by many social network scientists. To our knowledge, no such package which allows for the generation of graphs using both vertex attributes and network structure exists. Additionally, we innovated upon the original framework proposed by Sathanur et. al by augmenting the algorithm with an additional optional community label and creating a flexible framework that allows researchers to embed supplemental information about network structure (such as centrality measures, network geometry, etc.) into graph generation. Such generated networks can be distributed and analyzed without confidentiality concerns as they no longer contain potentially sensitive and identifiable real-world patient or physician data.

## Methods

### Property graph models with structural augmentation

Full details on the PGM procedure can be found in Sathanur’s original manuscript (Sathanur et al. 2017). To form the basis for the extensions developed herein, we specify notation and briefly review the PGM procedure as introduced in that manuscript. Let

$$\begin{aligned} G_s = <V_s,E_s,L, L(V_s)> \end{aligned}$$

be a property graph or network, where $V_s$ is the set of all vertices ($v_i$) and $E_s \subseteq V_s \times V_s$ is the set of all edges. $L=\{L_k\}^M_{k=1}$ is a set of M vertex label sets; and $L_k$ is the set of all possible values for the *k*th label such that $n_k=|L_k|$. Then $L(V_s)$ is the set of all label value vectors in 1-to-1 correspondence to $V_s$. Thus, each ${\bar{L}}(v_i)$ is drawn from the set of all possible joint label assignments $\mathcal{L}=\times_{k=1}^ML_k$ based on our label vector. There are $N=\prod _{k=1}^M n_k$ possible joint label categories. The *j*th joint label category is denoted $c_j$. The probability of drawing a joint label category $c_j$ is denoted $P_L(c_j)$ and defined as follows using the observed vertex labels in G:

$$\begin{aligned} P_L(c_j)=\frac{\sum _{i=1}^{|V_s|}1_{c_j}({\bar{L}}(v_i))}{|V_s|} \end{aligned}$$

which uses the following indicator function:

$$\begin{aligned} 1_{c_j}({\bar{L}}(v_i))={\left\{ \begin{array}{ll} 1,&{} \text {if } {\bar{L}}(v_i)=c_j\\ 0,&{} \text {otherwise} \end{array}\right. } \end{aligned}$$

Edge connectivity is modeled using a joint distribution over pairs of label categories $(c_j,c_{j'})$; which we denote $P_C$. This probability is calculated as:

$$\begin{aligned} P_C\left( c_j,c_j'\right) =\frac{\sum _{<v_i,v_i'\in E_s>}1_{c_j,c_j'}({\bar{L}}(v_i),{\bar{L}}(v_i'))}{|E_s|} \end{aligned}$$

using the following indicator function:

$$\begin{aligned} 1_{c_j,c_j'}({\bar{L}}(v_i),{\bar{L}}(v_i'))={\left\{ \begin{array}{ll} 1,&{} \text {if } \{v_i,v_i'\}\in E_s \text { and } \{{\bar{L}}(v_i),{\bar{L}}(v_i')\}=\{c_j,c_j'\}\\ 0,&{} \text {otherwise} \end{array}\right. } \end{aligned}$$

We then leverage $P_L$ and $P_C$ to generate a target graph, $G_T$ following Algorithm 1 as stated in Sathanur et al. (2017).

This procedure optimizes for the preservation of $P_L$, the proportion of vertices of each type, and $P_C$, the proportion of edges between each pair of vertex types, between the original and generated networks, provided the generated network is constructed with the same number of vertices and edges. In the case where the desired generated network has a different size from the original, $P_L$ and $P_C$ are preserved approximately due to rounding error.

To augment with degree, Sathanur et. al. proposed calculating vertex degree $\forall v_i \in G_s$, and assigning a new label by dividing the degree distribution into $n_b$ bins, where $n_b$ is tuned according to some error metric (for instance, the normalized root mean square error between the distribution of network statistics, motif-based measurements, etc.) between graphs. The degree augmentation algorithm is outlined in Algorithm 2 in Sathanur et al. (2017).

It has previously been shown that community detection as well as degree is beneficial in graph generation (Karrer and Newman 2010; Kirkley and Newman 2022). Thus, we sought to extend the above framework to consider a community detection augmentation option. To achieve this, we added additional labels $l_c$ to represent categorical, non-overlapping communities and appended these to the attribute labels *L*(*v*). These labels are then processed alongside the vertex and edge probabilities for attributes in $P_L$ and $P_C$. This framework is flexible; and hence allows users to add additional structural information as label categories by creating additional labels $l_{struct}$, adding these to $L_v$ and calculating subsequent node and edge probabilities. We call our method GeneRAtive Networks with Degree and Property Augmentation (GRANDPA). Algorithmically, we propose the additions to the PGM fitting procedure shown in Algorithm 1 (Sathanur et al. 2017).

**Table:** 

Algorithm 3 GRANDPA Algorithm: The extension of the PGM framework to consider a community detection augmentation option. The input to the algorithm is the generated graph $G_{X} = < V_{X} ,E_{X} ,L,L(V_{X} ) >$ from Algorithm 1 or Algorithm 2 as seen in Sathanur et al. (2017) and the number of vertices and edges in the target property graph $- n_{t} = |V_{T} |\;{\text{and}}\,\;m_{t} = |E_{T} |$.
1: **procedure** GRANDPA $( < V_{X} ,E_{X} ,L,L(V_{X} ) > ,\,n_{t} ,\,m_{t} )$ 2: Fit community detection model on the input graph and tune appropriately 3: **for** each $v \in V_{X}$** do** 4: Assign $l_{c} (v)$ value based on the community$(v)$ 5: Append the label vector $\bar{L}(v)\;{\text{with}}\;l_{c} (v)$ 6: **end for**
7: $error \leftarrow \infty$ 8: $G_{T} =$PGM-BASIC$( < V_{X} ,E_{X} ,L,L(V_{X} ) > ,\,n_{t} ,\,m_{t} )$ 9: $error \leftarrow computeError\left( {G_{S} ,G_{T} } \right)$ 10: **while** (*error* >* tolerance*)** do** 11: PGM-AUGMENTED$( < V_{T} ,E_{T} ,L,L(V_{T} ) > ,\,n_{t} ,\,m_{t} )$ 12: **end while** 13: **end procedure**

**Fig. 1:**
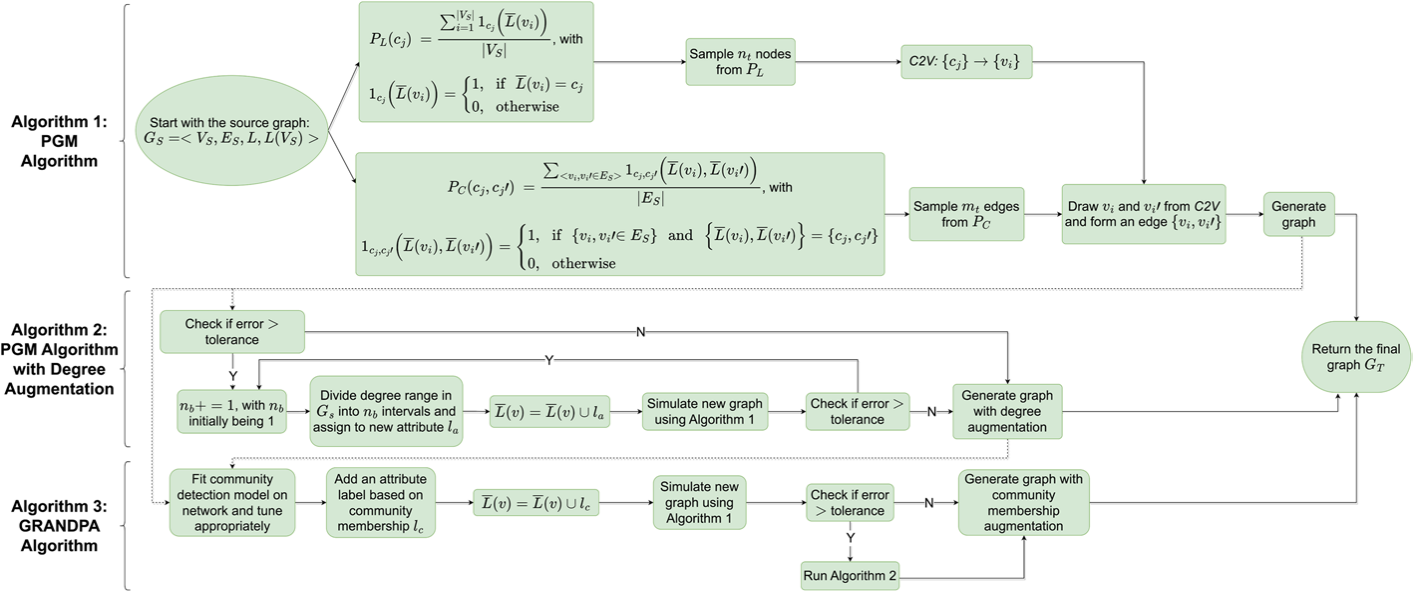
A depiction of the GRANDPA methodology used to generate graphs. **A** Shows an example original network, **B** shows a community calculation on this network, **C** and **D** show the generation of bags of possible vertices and nodes using $P_L(c_j)$ and $P_c$ respectively. **E** Demonstrates the sampling procedure, wherein edges are sampled without replacement. **F** is an example generated graph

The entire GRANDPA procedure is depicted in Fig. 1.

### GRANDPA: a user-friendly implementation in R

GRANDPA is available as an R package located at https://github.com/CarlyBobak/grandpa. GRANDPA depends exclusively on the *igraph* framework (Csardi 2014) and calculates probabilities using *tidyr* and *dplyr* Wickham et al. (2019). To use GRANDPA, users feed the function an *igraph* object, where any attributes which should be used to generate the joint label distribution should contain the word “label”. Thus, any vertex metric could be flexibly added to the label space to be used as part of the network generation. The source code was developed and analyses conducted in R version 4.1.1 (R Core Team 2019).

### Case study: Zachary Karate network

Contained within the *igraph* package is a small example network from Zachary’s 1977 paper studying conflict in a Karate network (Csardi 2014; Zachary 1977). We created two node category labels associated with network structure by using sampling weights to fix features whose distribution we wanted to retain while allowing for natural variation in the status of any given vertex and edge. The first label has three levels and its sampling weight is correlated with the degree distribution of the graph. For the second attribute, four categories are assigned depending on the distribution of closeness centrality. To easily assign sampling probabilities, we computed the quantiles of the degree distribution and closeness centrality scores as cutoffs, and assigned labels to nodes with varying sample probabilities based on those cutoffs. This sampling procedure ensures that the expected values of the concerned statistics are retained while allowing inherent noise between our assigned labels and the network structure, providing the opportunity to test whether attribute labels contribute to and are recovered by the networks constructed from our generative network modeling process when additional structural information is augmented.

To augment generated graphs with degree distributions, we tuned $n_b$ to be optimized over the values of 3, 5, 7, 10, and 15, and compared the normalized root mean square error (NRMSE) between the complementary cumulative distribution function (CCDF) of the vertex degrees between the original and the generated graphs. We also calculated communities in the original graph using the edge betweenness algorithm (Newman and Girvan 2004). We used the same algorithm to identify communities in GRANDPA-generated graphs, and systematically compared both the number and size of communities between the GRANDPA-generated graphs.

### Case study: unipartite medicare network

Bipartite networks can be constructed from insurance claims, such as those generated through the Centers for Medicare & Medicaid Services (CMS) Program (Landon et al. 2018). Networks connect patients to each physician they file a claim with over a designated period of time. Such networks can be projected to a unipartite space, where physicians are connected if they shared patients over a period of time (Barnett et al. 2011). To demonstrate the potential of GRANDPA in social network analysis for healthcare applications, we constructed a unipartite patient sharing network graph using Medicare Data from 2019, where two physicians are connected if they shared at least 11 patients. Such graphs are large, contain many nodes and edges, and are difficult to visualize (Allen et al. 2020). For demonstration purposes, we subset this graph by randomly selecting 3 physicians, finding all third-degree neighbors, and including those physicians and neighbors for our graph generation.

Each physician in our data had a known primary medical specialty. We calculated community membership for each physician based on hospital affiliation, working groups, geographical regions, etc. using the fast-greedy algorithm in igraph (Csardi 2014; Clauset et al. 2004). Additionally, we calculated the linchpin centrality of each physician based on their connections to communities outside their own (Nemesure et al. 2021), and binned linchpin centrality into labels with no centrality (=0), low centrality (between 0 and 0.2), or high linchpin centrality ($\ge 0.2$). We approximately augmented degree by forming bins with intervals of 5 (i.e., the first bin was from 0 to 4, the second from 5 to 9, and so on).

To compare graphs, we plotted the CCDF of the degree and eigenvector centrality distributions, calculated the NRMSE between the original source graph and the generated graphs, and performed a Kolmogorov–Smirnov (KS) test to evaluate if the observed measurements originated from different distributions (Sathanur et al. 2017; Massey 1951). We also ran the fast-greedy community detection algorithms on the generated graphs and optimized for graphs which approximated both the size and number of generated communities. To compare community organization between graphs we used propensity matching to identify vertex matches between the original graph and the generated graphs using the *MatchIt* package in R (Ho et al. 2011). To overcome the label switching problem, for each generated graph we used the k Nearest Neighbours (Cover and Hart 1967) clustering algorithm to match vertices such that categorical labels were matched exactly to find the closest vertex matches. We then calculated a community agreement score corresponding to the number of vertices with correct community membership over the total number of vertices, or:

$$\begin{aligned} \frac{|\{v_i~|~l_c(v_i)\in G_T =l_c(v_i)\in G_s\}|}{|V_s|} \end{aligned}$$

We ran a binomial regression on the original graph which aimed to predict if a physician’s primary specialty was hospitalist or internal medicine using a homophily variable (same specialty). We repeated this analysis on our best-generated graphs, and compared the returned coefficients (odds ratios) to evaluate if models built on the simulated graph reflect the results of models built on the original.

## Results

We use two real-world networks to demonstrate the utility of the GRANDPA algorithm, particularly in graphs with inherent community structure. The first is the canonical Karate network discussed by Zachary (1977). The second is a unipartite patient-sharing network which links healthcare providers who shared Medicare patients in 2019.

### Case study: Zachary Karate network

To illustrate the generality of our GRANDPA algorithm, we first chose the network of a university karate club. The karate network consists of 34 vertices with 78 undirected edges. Each node represents a club member, and an edge between two members indicates their interaction.

**Fig. 2:**
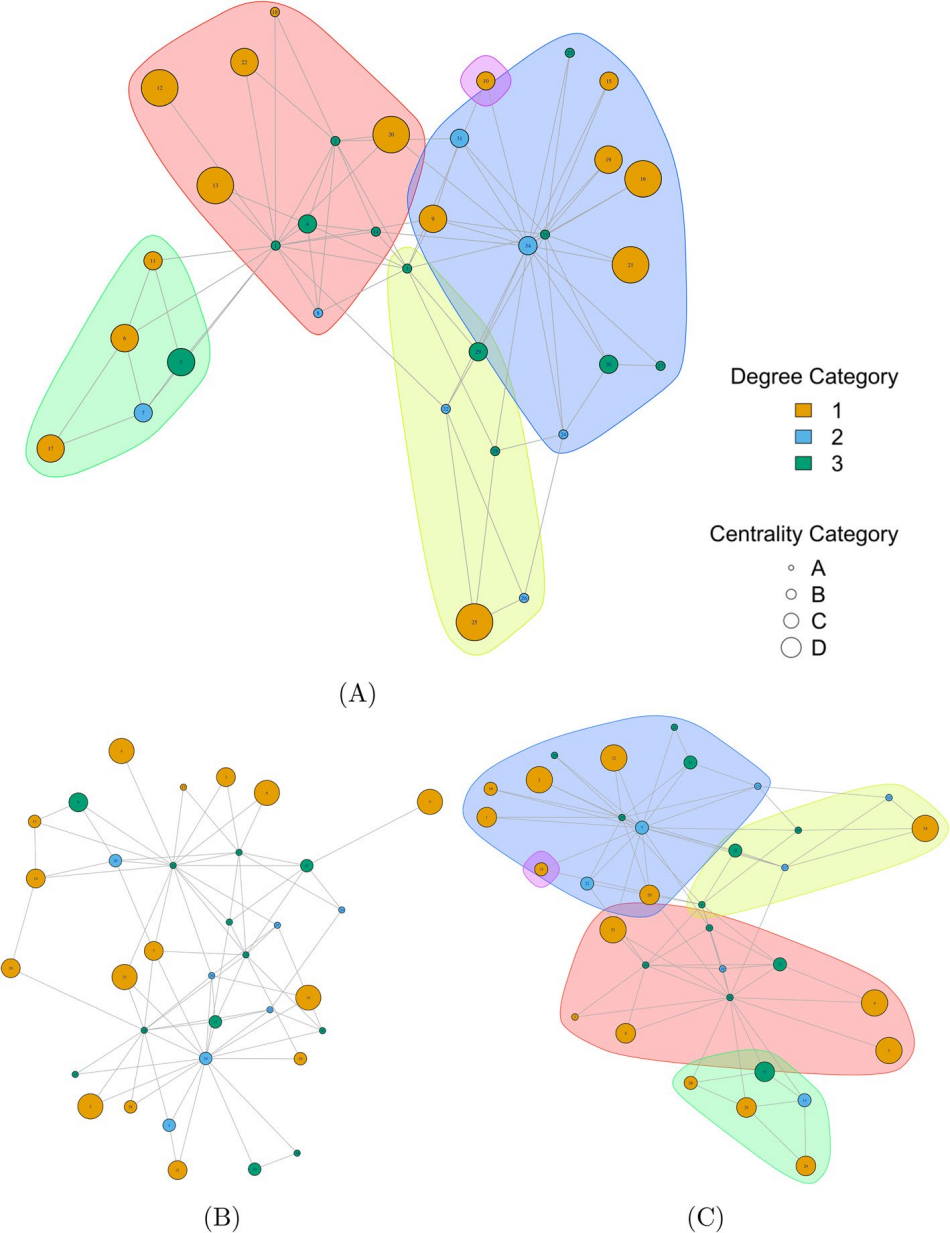
The original and generated graphs from Zachary’s Karate network (Zachary 1977). **A** The original graph, where vertex color indicates a category associated with degree, vertex size represents a category associated with centrality, and community areas are drawn. **B** The PGM-generated graph, if just categorical attributes and degree augmentation are used to generate the network. **C** The GRANDPA-generated graph, where attributes, community labels, and degree are used to generate the network. In Fig. 2**C**, we recaptured similar vertex attribute relationship and nearly identical communities in both overall number of communities, size of communities, and internal community structure

The plots of the three selected graphs are shown in Fig. 2. To better visualize the correlation between network structure and node attributes, we selected colors to depict the different levels of the first label, and sized nodes to show the corresponding categories of the second one. The original graph, with generated labels and communities, is shown in Fig. 2A.

**Table 1:** Comparison of the degree distribution NRMSE between the original Zachary’s Karate Network, and the Karate network recovered using the PGM models or the GRANDPA model with community augmentation over the nbins parameter

	PGM	PGM with degree augmentation	GRANDPA with degree and community
$n_b=3$	0.187	0.041	0.042
$n_b=5$	0.187	0.048	0.024
$n_b=7$	0.187	0.028	0.024
$n_b=10$	0.187	0.048	0.000
$n_b=15$	0.187	0.031	0.000

Results from tuning $n_b$ using both a PGM algorithm with degree augmentation, and then GRANDPA algorithm with degree and community augmentation are shown in Table 1. As perfect recovery of the degree distribution occurred in the community model with 10 bins, we set $n_b=10$ for our final plots.

**Fig. 3:**
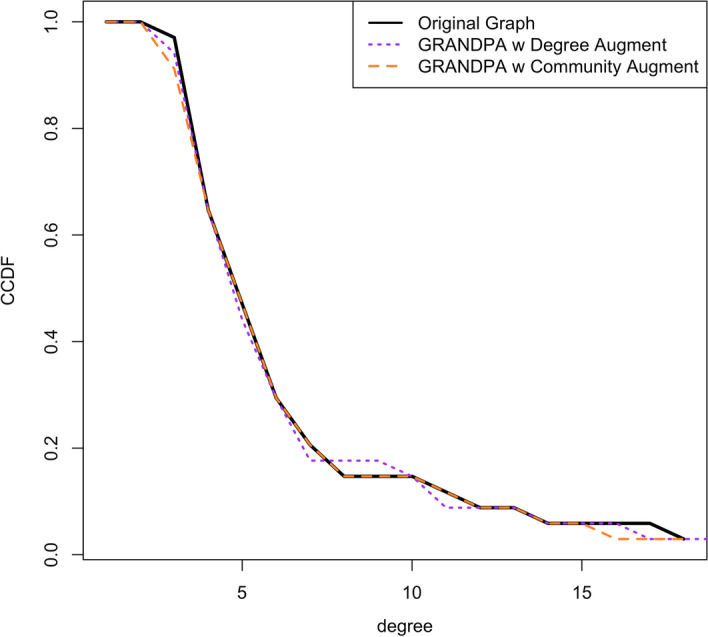
The CCDF of the vertex degree between the original and generated networks for Zachary’s Karate Club network (Zachary 1977)

Two generated graphs are shown. The first, in Fig. 2B, represents a PGM-generated graph with both attributes and augmented degree ($n_b$=10). The overall degree distribution across the vertices is close to the original graph with $NRMSE=0.161$ and shown in Fig. 3. However, visually it is clear that the community structure present in the original graph is missed in the PGM-generated graph.

When we create a community label following the marked areas and feed this to the GRANDPA algorithm, we quickly recover a graph that is highly similar to the original (Fig. 2C). The NRMSE between the degree distribution of the original and GRANDPA-generated graph with community augmentation is nearly identical, as shown in Fig. 3 with overall value $NRMSE=0.0508$. As well, we recaptured nearly identical communities, in both overall number of communities, size of communities, and internal community structure.

### Case study: medicare unipartite physician network

**Fig. 4:**
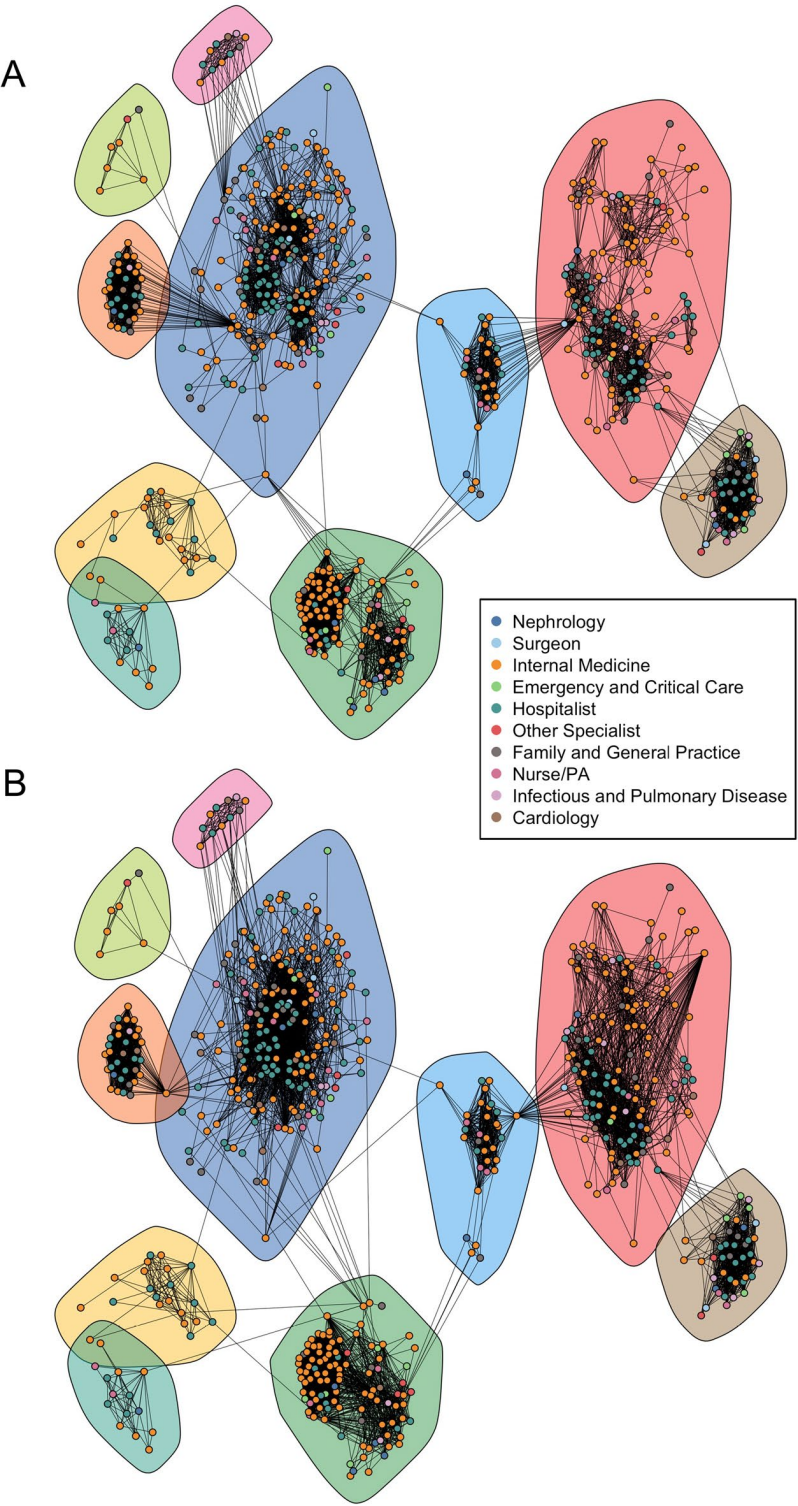
The original and GRANDPA-generated graphs from our 2019 Medicare data. **A** The original graph, where vertex color indicates a physician’s primary specialty. **B** The final GRANDPA-generated graph with community, degree, and linchpin centrality augmentation. In Figure 4**B**, we recovered the same number of communities and similar in-between and internal community structures compared to Figure 4**A**

We sought to test our algorithm on a large, heterogeneous healthcare network which is more representative of those used in biomedicine and healthcare policy studies. We constructed a network using Medicare claims data from 2019 (8021 vertices; 82,893 edges) and subset it to include 608 physicians (vertices) and 6480 patient-sharing connections (edges). The original graph is shown in Fig. 4A. Vertex colours are representative of the physician’s primary specialty. Community-level clustering is present, and reflective of hospitals, geographic regions, and physician working groups. A fast-greedy clustering algorithm detected 10 disparate communities with sizes $\{209,134,105,41,34,32,21,14,11,7\}$.

**Fig. 5:**
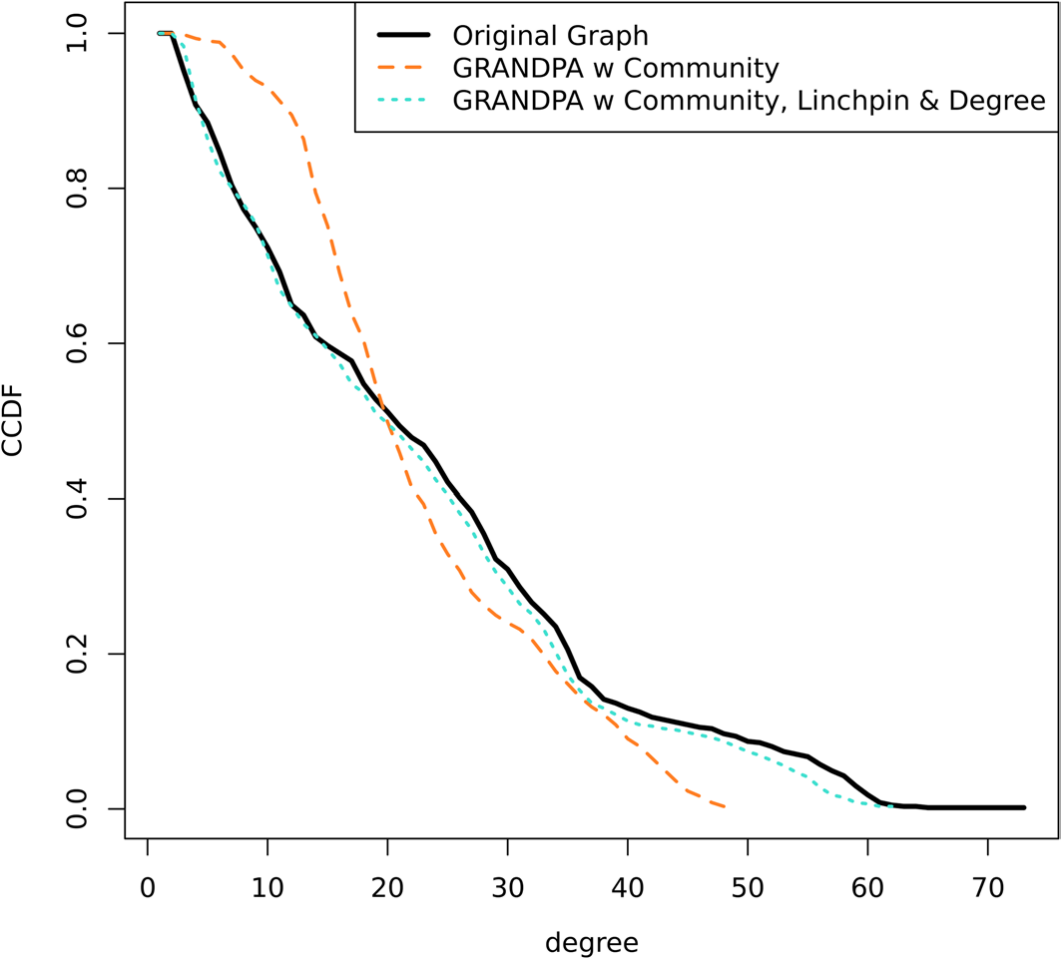
The CCDF of the vertex degree between the original and generated networks for the 2019 Medicare Data from CMS

**Table 2:** Comparison of network measures between the original CMS subgraph and various generated graphs

	Degree		Eigenvector centrality		Community agreement
	NRMSE	KS (*p* value)	NRMSE	KS *p* value	
PGM (specialty)	0.404	0.387 (< 0.001)	0.167	0.901 (< 0.001)	0.039
PGM (specialty and degree)	0.045	0.069 (0.109)	0.167	0.873 (< 0.001)	0.039
GRANDPA (specialty and community)	0.237	0.248 (< 0.001)	0.165	0.543 (< 0.001)	1.000
GRANDPA (specialty, community and degree)	0.054	0.038 (0.777)	0.088	0.173 (< 0.001)	0.993
GRANDPA (specialty, community, degree and linchpin)	0.051	0.033 (0.897)	0.028	0.102 (0.003)	0.990

Normalized root mean square error (NRMSE) and Kolmogorov–Smirnov (KS) test *p* values were calculated to evaluate the distribution of degree and centrality measures across the generated graphs

Propensity matching at the vertex level was conducted between the original and generated graphs to calculate community agreement

Graphs were generated using just attribute-level information (Specialty), attribute information with degree augmentation, attribute information with community augmentation, and attribute information with community augmentation and degree augmentation. Similar to the Karate Case Study, PGM-generated graphs without community augmentation lacked the community structure observed in the original graph. While the GRANDPA-generated graph with only attribute information and community augmentation produced a graph with matching communities, the degree distribution was poorly recovered (NRMSE=0.237; Fig. 5). A Kolmogorov–Smirnov (KS) test rejects that the observed degrees originate from the same distribution as the original physician network. Augmenting further with a degree label ($n_b=15$) reduced the NRMSE to 0.054 and a KS test statistic under which the null hypothesis of equal distributions is no longer rejected. Full comparisons of network characters across all generated graphs can be seen in Table 2. However, the GRANDPA-generated graph did not contain key characteristic nodes connecting communities [often referred to as “bridge” nodes (Ezeh et al. 2019)] as was observed in the original graph.

To attempt to ameliorate the lack of recovery of bridge nodes, we used the Linchpin Centrality score (Nemesure et al. 2021) to identify nodes which are ‘one-of-a-kind’ compared to their neighbors’ community labels, and further augmented our GRANDPA algorithm with a label corresponding to linchpin centrality. The GRANDPA-generated graph with community, degree and linchpin centrality augmentation is shown in Fig. 4B. The NRMSE between the CCDF of the final graph and the original was 0.051 (Fig. 5) and similarly the KS-test indicates that the observed degrees are unlikely to have arisen from the same distributions and thus model for the graph. Similar to the original graph, the final graph had 10 identifiable communities with sizes $\{205,132,108,42,35,33,21,14,11,7\}$, showing a high degree of concordance with the community structure of the original graph. Mapping similar vertices between the original graph and the generated graph to compare community membership indicates a community agreement fraction of 0.99, wherein 6 vertices out of 8021 were separated into different neighbouring communities between the original and returned graph.

We used a multi-criterion decision rule to select the final generated graph which sought to optimize over the degree distribution, community accuracy, and by a comparison of visual features across graphs. To this end, we prefer the final GRANDPA generated graph which used a specialty label to generate graphs with the same proportion of vertices of each type and proportion of edges with each pair of vertex types as nodes and also augmented with degree, community and linchpin centrality to recover additional salient elements of the original graph.

**Fig. 6:**
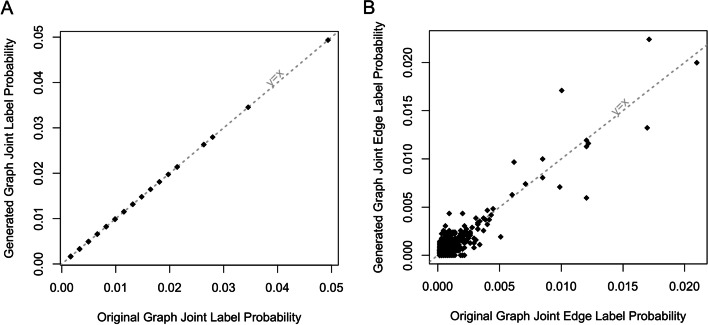
The calculated joint label probabilities between the original physician graph based on actual Medicare claims data and final GRANDPA generated graph. **A** Shows the returned vertex joint label probabilities and **B** shows the edge label probabilities

By default, the GRANDPA algorithm seeks to preserve the joint label probabilities between graphs. To demonstrate this, we calculated the joint label probabilities on the original and generated graphs. In every case, the joint vertex label probabilities are preserved exactly. Occasionally, the random sampling occurs such that the joint edge label probabilities cannot be matched exact without the occurrence of self-connections or duplicated edges, resulting in slightly inaccurate edge label probabilities. This occurs in our final model where we generated a graph using specialty, degree augmentation, community augmentation, and linchpin centrality. We calculated this error rate by considering the sum of square residuals from a perfect recovery, which in this case is small at 0.0004. This relationship is shown in Fig. 6. This process was repeated on the entire CMS network which likewise essentially preserved the vertex join label probabilities exactly, but had a sum of square residuals of $2.92 \times 10^{-5}$.

**Table 3:** Logistic Regression results for modelling if a physician’s specialty was classified as a care coordinator (hospitalists and internal medicine) or other specialist given the percentage of neighbours with the same specialty across the original generated networks

	Intercept			Same specialty			AIC
	Odds ratio	95% CI	*p* value	Odds ratio	95% CI	*p* value	
Original	0.063	(0.033, 0.111)	< 0.001	1.116	(1.096, 1.142)	< 0.001	138.61
PGM (specialty and degree)	< 0.001	(0.000, 0.004)	< 0.001	1.386	(1.218, 1.937)	0.001	17.10
GRANDPA (specialty, community and degree)	0.004	(< 0.001, 0.017)	< 0.001	1.203	(1.152, 1.286)	< 0.001	42.88
GRANDPA (specialty, community, degree and linchpin)	0.0235	(0.009, 0.053)	< 0.001	1.139	(1.113, 1.174)	< 0.001	93.98

We sought to evaluate if models trained on the GRANDPA-generated graph were reflective of results trained on the original graph. To this end, we trained a regression model on both the original graph and the final graph (Fig. 4) which aimed to predict if a physician’s primary speciality was either hospitalist or internal medicine given the proportion of their neighbors with the same specialty. In the original graph, the odds ratio that a physician is a hospitalist or internal medicine doctor is 1.116 (95% confidence interval: 1.096, 1.142) when the proportion of physician neighbors within the same specialty increases by 1%. In the final GRANDPA-generated network, the same odds ratio as in the original graph of 1.139 obtained (95% confidence interval: 1.113, 1.174); demonstrating satisfactory recovery of the true odds ratio. Full regression results across the generated networks are shown in Table 3 and demonstrate that the regression model fit on the final GRANDPA generated graph best recovered the model output fit on the original graph. Two regression models estimated on the generated networks did not converge and are omitted from the presentation of these results. Ten GRANDPA generated graphs were constructed that included specialty, community, degree, and linchpin labels; these were determined using different random seeds and so we recalculated the odds ratio for each graph. The mean odds ratio for having the same specialty in the GRANDPA-generated graphs was 1.143 with a standard deviation of 0.011.

## Discussion

Data sharing and availability are fundamental to reproducibility of healthcare research and trust in the results obtained. However, concerns on protecting patient privacy need to be balanced (Wirth et al. 2021; Clayton et al. 2019; Hammack-Aviran et al. 2020; McGraw and Mandl 2021). This is also true for the analysis and distribution of networks generated using patient health data. The American Medical Association recently surveyed patients on the use of their medical data for research, and 75% of respondents wanted the ability to opt-in for research use. As well, 92% of respondents felt that their data should not be available for purchase by either corporations or individuals (Medical Association 2022). Separately, Hammack-Aviran et al. noted in a qualitative survey that patients would prefer transparency and choice in having their electronic medical records included in research efforts, often wanting information prior to consent on all research objectives and stakeholders (Hammack-Aviran et al. 2020). Moreover, open-source software is often desirable for analyzing patient data, but may have security vulnerabilities which violate HIPAA compliance (Farhadi et al. 2019). Assessing open-source software for compliance is a non-trivial task (Farhadi et al. 2019). Researchers are required to de-identify data prior to public release. However, in the case of biological data, such as data generated from DNA, data itself is a unique identifier (Clayton et al. 2019). Moreover, patients often view their genetic and molecular data to be private (Clayton et al. 2019). Patient trust is a necessary component in healthcare research, and hence the distribution and analysis of networks with embedded patient data needs to be done with extreme care. Simulation of networks can alleviate many of these concerns as it allows for the creation of networks which maintain macro-level relationships without revealing any individual-level observations.

Hence, when generating graphs developed on sensitive or private data, we aimed to retain attribute values of vertices in such a way that they do not identify the original actor. Thus, we sought to generate a graph which retains network properties as opposed to partially retaining ‘real’ actors. Techniques such as Attribute Inference Attacks may be beneficial in demonstrating that combinations of identifiable labels are not present in generated networks (Gong and Liu 2018).

In this work, we demonstrated that GRANDPA can be used to generate both simple and complex graphs which are highly representative of original real-world networks. The GRANDPA framework allows the implementation of a family of methods, in which researchers can customize information pertaining to attributes and network structure to generate realistic graphs. Both case studies reproduced graphs with highly similar community structure and degree distributions while recovering the relational attribute structure by design. In our two case studies, degree distributions were highly preserved, and community detection algorithms identified nearly identical communities between the original and GRANDPA-generated graphs.

Our case study of Zachary’s karate network reproduced a graph that is highly representational of the original graph, with many motifs within communities preserved between the original and GRANDPA-generated graphs. Our case study of a patient-sharing network generated from CMS data likewise demonstrated that community structure could be preserved, and also that regression coefficients generated on the original and generated graphs overlapped, suggesting that models fit on the generated graphs are reflective of real-world findings.

We demonstrated that the GRANDPA algorithm can flexibly be augmented for not only degree and community structure, but also for vertex positional network features such as centrality measures. Indeed, generated graphs are only as accurate as their underlying models, and improvements in comparisons of graph topology may be possible with additional augmentation.

In Table 2 we demonstrated strong performance with the GRANDPA algorithm that considered Speciality, Community, Degree, and Linchpin labels. This algorithm demonstrated the strongest performance across the degree distribution in terms of the eigenvector centrality NRMSE and the KS statistics while maintaining strong performance across the community agreement and degree distribution NRMSE. In some cases researchers may prioritize optimizing some accuracy measures over others with no one method being uniformly the best overall accuracy measures. This is a frequently observed phenomenon in modeling and machine learning (Wolpert and Macready 1997). Researchers should be sure to specify models such that they optimize over the network similarity measures of most relevance to the given setting or study.

This was evidenced by our slightly biased estimate of the odds ratio of a physician being in internal medicine or classified as a hospitalist based on their proportion of same speciality neighbors. The bias in the estimator is likely due to a loss of information between the original graph and generated graphs, where medical specialty was the only directly measured attribute used to generate the graph, which may have over-emphasized the importance of connections based on medical specialty (e.g., if medical speciality is correlated with other measures that are also partially responsible for the generation of the graph). Researchers generating complex graphs should take care to confirm that the combination of provided labels adequately represents the target relationships of interest.

As well, the current framework weights all attribute and structural augmentation labels equally. Future directions will include optimizing a weighting function of possible labels using a regression framework in order to best capture information related to both network structure and vertex attribute relationships.

## Conclusions

Graphs generated from real data have many possible use cases. Scalable graphs can be important for bench-marking tools and graphs generated from confidential data can be safely distributed with software and publications. Moreover, the distribution of generated biomedical graphs may reduce the costs of data acquisition for new researchers, allow greater access for trainees, reduce the risk of analyzing data with novel tools that violate attempts to analyze the data securely, provide possible datasets for validation studies, support reproducible research, and be useful in pilot analyses for hypothesis generation. GRANDPA is a flexible and user-friendly framework which will allow researchers across disciplines to generate meaningful graphs from real data when the original graph cannot be shared with them. We created an open-source and freely distributed R package accompanying the GRANDPA algorithm so that researchers can easily and generate graphs with a wide range of network features underlying them. As is true in any model building exercise; researchers should take care to include appropriate variables and select appropriate network similarity measures to optimize the recovery of their source graph.

## Data Availability

Original data are available on request due to privacy or other restrictions. Simulated data is available at https://github.com/CarlyBobak/grandpa.

## Abbreviations

ATG: Attribute graph model
CCDF: Complimentary cumulative distribution function
CMS: Centers for medicare and medicaid services
ERGM: Exponential random graph model
MAG: Multiplicative attribute graph
GRANDPA: Generative networks using degree and property augmentation
NRMSE: Normalized root mean square error
KS: Kolmogorov–Smirnov
PGM: Property graph models
